# ID 240 - Análise Retrospectiva da População Elegível e Impacto Orçamentário de Recomendações sobre Medicamentos Submetidos à Consulta Pública na Saúde Suplementar em 2022 e 2023

**DOI:** 10.5327/2237-9622.2025.v34s1.240

**Published:** 2025-11-25

**Authors:** Cecília Farinasso, Rafael Leite Pacheco, Cecília Menezes Farinasso, Ana Luiza Cabrera Martimbianco, Camila Monteiro Cruz, Carolina de Oliveira Cruz Latorraca, Isabela Porto de Toledo, Patrícia do Carmo Silva Parreira, Roberta Borges Silva, Verônica Colpani, Rachel Riera

**Keywords:** análise de impacto orçamentário, acesso a medicamentos essenciais e tecnologias em saúde, saúde suplementar, ANS

## Abstract

**Introdução:**

O Núcleo de Avaliação de Tecnologias em Saúde/Núcleo de Evidências do Hospital Sírio-Libanês (Nats/NEv-HSL) atua em colaboração com a Agência Nacional de Saúde Suplementar (ANS) na avaliação crítica de propostas para atualização do Rol de Procedimentos e Eventos em Saúde. Parte dessa avaliação inclui a reanálise de impacto orçamentário (AIO) projetado com as tecnologias em avaliação pela ANS. Logo, o objetivo deste trabalho foi analisar a média da população elegível e o impacto orçamentário acumulado em cinco anos das análises realizadas pelo Nats/NEv-HSL em 2022 e 2023.

**Método:**

Estudo descritivo conduzidos no Nats/NEv do Hospital Sírio-Libanês. Os desfechos incluíram número médio de pessoas elegíveis na AIO; proporção de pacientes elegíveis por 100 mil usuários da saúde suplementar; impacto orçamentário acumulado em cinco anos; e impacto orçamentário médio anual por paciente elegível. As fontes de informações foram os documentos submetidos pela equipe técnica da ANS à consulta pública referentes a medicamentos entre janeiro de 2022 e junho de 2023. Os dados foram extraídos manualmente por dois pesquisadores, e a análise foi realizada pelo software Stata® (versão 17). O protocolo está disponível em: https://osf.io/srd7g.

**Resultados:**

Foram identificadas 50 análises técnicas submetidas à consulta pública, sendo 37 (74%) elegíveis. A mediana de pacientes foi 480, variando de 53,8 a 117.996 pessoas. Já a proporção de pacientes elegíveis foi de 0,896 pacientes por 100 mil usuários da saúde suplementar, variando de 0,1 a 220,2 por 100 mil usuários. Em análise de subgrupo para condições oncológicas graves, todas as análises de medicamentos antineoplásicos tiveram proporção estimada em menos que 10 pacientes elegíveis por 100 mil usuários. O impacto acumulado das recomendações favoráveis foi de R$ 4.213.912.839,86 (R$ 210.695.641,99 por trimestre), e o impacto anual por paciente elegível seria de R$ 12.239,64. Já para recomendações não favoráveis, o impacto orçamentário esperado em cinco anos seria de R$ 1.412.301.368,02, com impacto anual por paciente elegível de R$ 2.223,26. As recomendações favoráveis após a análise técnica projetaram economia em 36% dos casos (n=10, total n=28), garantindo acesso a tratamento a cerca de 70 mil pacientes a cada ano.

**Conclusão:**

No que se refere ao cálculo de população elegível, as análises contemplaram majoritariamente pacientes graves e/ou oncológicos, com proporção abaixo de 65 pessoas por 100 mil usuários da saúde suplementar. Quanto ao impacto orçamentário projetado, este correspondeu a 0,37% da despesa assistencial no primeiro trimestre de 2023, e as análises técnicas contribuíram para a manutenção da eficiência da saúde suplementar.

